# Virtual Anatomy Classes among the First and Second Year Medical and Dental Students of a Medical College: A Descriptive Cross-sectional Study

**DOI:** 10.31729/jnma.6575

**Published:** 2021-08-31

**Authors:** Sarbada Makaju, Chandra Kala Rai

**Affiliations:** 1 Department of Anatomy, Kathmandu Medical College and Teaching Hospital, Duwakot, Bhaktapur, Nepal; 2 Department of Physiology, Kathmandu Medical College and Teaching Hospital, Duwakot, Bhaktapur, Nepal

**Keywords:** *anatomy*, *embryology*, *medical students*

## Abstract

**Introduction:**

The pandemic of COVID-19 affected every single person in the world. This pandemic also affected the academics of medical and dental colleges of Nepal. In these conditions, the virtual study was used as an emergency measure during the COVID-19 period, with an adaptation to the "new normal" to deliver preclinical medical education. This has brought both challenges and opportunities to medical education. The objective of the study is to find out whether the anatomy virtual classes were helpful among the first and second year medical and dental students of a medical college.

**Methods:**

The descriptive cross-sectional study was conducted on 206 first and second-year medical and dental students of a medical college between 1st May 2021-31st May 2021 after getting the ethical clearance from the Institutional Review Committee (reference no. KMC-IRC 0504202109). The convenient sampling was done. The data were analyzed with Statistical Package for the Social Sciences 20 version. Point estimate at 95% Confidence Interval was calculated along with frequency and percentage for binary data.

**Results:**

Out of the total 206 students, 173 (84%) (78.99-89.01 at 95% Confidence Interval) found that virtual anatomy classes were not helpful for the study of the anatomy classes.

**Conclusions:**

From the study, we conclude that more than half of the students found that virtual classes were not helpful for the study of anatomy classes. Students have difficulty in studying dissection, cadaveric, and embryological structures via virtual classes. Physical class is better for studying anatomy than virtual classes for medical and dental students.

## INTRODUCTION

At the end of December 2019, a new virus emerged from Wuhan China named COVID-19. It is a respiratory disease that can transmit through inhalation. As the day passed, the mortality rate was increased.^1^ In our country, the first case was reported on 13/1/2020 and confirmed on 23/1/2020^2^

On 24/03/2020 first lockdown was announced by the Nepal government after finding the second case of COVID-19 in Nepal. All the mobility of the people was halted except the emergencies and medical shops. The educational sectors were closed completely.^3^ That situation was challenging for the medical and dental academics in which Anatomy is a pillar for clinical skills.^4-6^ During this pandemic, the theory lectures had to switch to digital.

The objective of the study was to find out whether the anatomy virtual classes were helpful among the first and second year medical and dental students of a medical college.

## METHODS

The descriptive cross-sectional study was carried out in the first and second year medical and dental students of Kathmandu Medical College and Teaching Hospital between 1^st^ May 2021- 31^st^ May 2021 after getting the ethical clearance from the Institutional Review Committee (reference no. KMC-IRC 0504202109). The data was collected from the 23rd and 22nd batch of medical and 7th, 8th batch of dental students of Kathmandu Medical College and Teaching Hospital. All the interested students were included after taking the three months virtual classes of First and Second year medical and dental students. The students who were chronically absent in the virtual class during three months period were excluded. Participants were enrolled using convenient sampling.

The sample size was calculated by using formula:

n = Z^2^ × p × q / e^2^

  = (1.96)^2^ × (0.50) × (1-0.50) / (0.07)^2^

  = 196

Where,

n = required sample size
Z = 1.96 at 95% Confidence Interval (CI)
p = prevalence for maximum sample size, 50%
q = 1-p
e = margin of error, 7%

Here, the calculated sample size is 196. We have included 206 responses from the medical and dental students for this study.

The participants were included after taking verbal informed consent among first and second year medical and dental students in Kathmandu Medical College and Teaching Hospital between 18-20 years by using convenient sampling. Each student was explained the objectives of the study via Viber or e-mail. A multiple-choice close-ended questionnaire was designed through Google forms and sent it through the Viber or email of the students. The questionnaire was completely based on the virtual learning activities in the field of Anatomy. The student's feedback was taken after more than 3 months of digital anatomy education. The feedbacks of the students were kept completely confidential and anonymous. The data were analyzed with Statistical Package for the Social Sciences 20 version.

## RESULTS

Out of the total 206 students, 173 (84%) (78.99-89.01 at 95% Confidence Interval) found that virtual anatomy classes were not helpful for the study of the anatomy classes. Among them, 106 (61.3%) were friendly enough to handle gadgets for virtual learning, 57 (32.9%) were not friendly while 10 (5.8%) were not sure.

About 148 (85.5%) students found it difficult to understand embryology without the model. Nearly 143 (82.7%) can't co-relate the structures through the online classes (Table 1).

**Table 1:** Responses among the students who find that virtual anatomy classes were not helpful (n = 173).

	Yes n (%)	No n (%)	Maybe n (%)
During the early stage of lockdown are you aware of COVID-19 will be a pandemic?	142 (82.1)	30 (17.3)	1 (6)
During the early stage of lockdown are you aware that COVID-19 may be an endemic	75 (43.4)	63 (36.4)	35 (20.2)
During the early stage of lockdown do you friendly enough to handle computer/laptop/Electric gadgets for virtual learning?	106 (61.3)	57 (32.9)	10 (5.8)
Do you feel a lack of proper gadgets, high bandwidth, and strong internet connections; barriers in your current learning process?	127 (73.4)	24 (13.9)	22 (12.7)
Do you miss the dissection and cadaveric studies in Anatomy?	138 (79.8)	35 (20.2)	
Do live dissections help you to make the subject easy to grasp comparatively?	155 (89.6)	18 (10.4)	
You have gross theory online without the aid of real cadaveric dissection classes. Is it effective?	11 (6.4)	162 (93.6)	
Is microscopic anatomy tough to understand without spotting slides under microscopy?	143 (82.7)	30 (17.3)	
Do you find it difficult to understand embryology without a model?	148 (85.5)	25 (14.5)	
Are you satisfied with the current assessment system?	37 (21.4)	134 (77.5)	2 (1.1)
Do you feel distracted by home comforts/discomforts?	136 (78.6)	37 (21.4)
Do you find any difficulty in time management?	147 (85)	26 (15)
Do you feel a lack of self­motivation in the current scenario?	146 (84.4)	27 (15.6)
Do you miss your real college environment, companions, cultural and sporting events?	162 (93.6)	11 (6.4)
Does your imagination, concept was crystal clear through virtual studies of Anatomy?	11 (6.4)	162 (93.6)
Do you think the lectures should run hand in hand with the topic of dissection?	164 (94.8)	9 (5.2)
Do the anatomy lecturer was friendly through visual online?	78 (45.1)	95 (54.9)
Is it difficult to co-relate the structures through the online classes?	143 (82.7)	30 (17.3)

Among 173 students, S7 (50.3%) used smartphones, 77 (44.5%) used laptop and 9 (5.2) used Ipad (Table 2).

**Table 2:** Gadgets used by students for learning.

Gadget used	n (%)
Ipad	9 (5.2)
Laptop	77 (44.5)
Smartphone	S7 (50.3)

For virtual classes, 90 (52%) perefer PowerPoint presentations, 2 (1.15%) prefer presentation with video recording and 79 (45.7%) prefer video recording of online classes (Table 3).

**Table 3:** Student preferred modes of learning.

Mode of learning	n (%)
Powerpoint presentations	90 (52.0)
Physical classes	2 (1.15)
Presentation with recorded video of classes	2 (1.15)
Video recorded	79 (45.7)

## DISCUSSION

The COVID-19 pandemic has brought drastic changes in the lifestyle of human beings. All the routines were completely changed. These changes had to adapt to survive from COVID-19 pandemic. All the physical classes were shut down and turn into virtual classes. As a consequence of the COVID-19 pandemic, physical studies were suspended, and a rapid transition to virtual learning occurred. In our study, 173 (84%) find that virtual classes didn't help to study anatomy classes. According to some authors, when students lost access to dissection rooms, they lost access not only to cadavers, but also to a range of other optimal learning modalities: prosections, models, pathology specimens, skeletons, and others.^7,8^ This theory was completely accepted by this study. In this study, 79.8% of students missed the dissection and cadaveric studies.

Nitasha, et al. came with the result that 87 (50.3%) used the smartphone while taking the online classes which supported the result of this study (50.3%).^9^

The virtual study was used as an emergency measure during the COVID-19 period, with an adaptation to the "new normal" to deliver preclinical medical education, which has brought both challenges and opportunities to medical education.^10^ The study done by Kreeti, et al. found that students prefer active and engaging learning strategy for Anatomy which support this studies.^11^ In this study, the students understand more in physical classes than the virtual ones.

The majority (65%) of them missed every aspect of anatomy education i.e., cadaveric lab, face-to-face lectures which support this study.^6^ These studies proved that the students missed the cadaveric dissection study 138 (79.8%). This also proved that students preferred to study Anatomy with the aid of Cadaveric live dissection.

This study was carried out in a limited sample size. The participants were enrolled using a convenient sampling technique. Therefore, this result may not be generalized to medical and dental students all over Nepal. Further studies should be carried out with a larger sample that will representative of medical students all over Nepal.

## CONCLUSIONS

From the study, we conclude that more than half of the students find that virtual classes were not helpful for the study of anatomy classes. Students have difficulty in studying dissection, cadaveric, and embryological structures via virtual classes. Physical class is better for studying anatomy than the virtual classes for medical and dental students. Therefore, virtual classes were an option but not a permanent solution during this pandemic.
